# A commentary on ‘Global research status and frontiers on autophagy in hepatocellular carcinoma: a comprehensive bibliometric and visualized analysis’

**DOI:** 10.1097/JS9.0000000000001343

**Published:** 2024-03-27

**Authors:** Sike He, Hao Zeng

**Affiliations:** Department of Urology, Institute of Urology, West China Hospital, Sichuan University, Chengdu, People’s Republic of China

*Dear Editor,*

Recently, we read the article published in the *International Journal of Surgery* by He and colleagues^1^ with great interest. This present bibliometric analysis systematically and comprehensively presents the current research status, future hotspots, and frontiers of autophagy in hepatocellular carcinoma (HCC) by analyzing the trends in publication, distribution of countries and institutions, co-cited networks (author, journal, and reference), and keywords. Since growing evidence confirmed that autophagy plays a role in cancer biology, this bibliometric analysis is much needed and is useful to provide an overview of this special topic^2^. We sincerely congratulate the authors for their outstanding contribution to this research field. After carefully reading, here we would like to express some of our views that may help enhance this area for further research.

First, the meticulous arrangements of search strategies play a key role in bibliometric analysis. He *et al*. made the search strategy rigorous by using the combination of synonyms of HCC to further reduce the omission of relevant literature and maximally avoid bias, which is a useful example for bibliometric analysis. The medical subject heading (MeSH) terms provided in PubMed can allow searches to cover a broader range of literature since MeSH contains a hierarchical structure and MeSH descriptors represent different semantic types, which should be recommended in bibliometrics^3^. Second, the keyword analysis is significantly meaningful since the authors completed the integration of keywords to illustrate the core concepts of the studies. Simple keywords displayed by articles are sometimes over-generalized, failing to provide sufficient information. The keywords clusters visualization is worthy of highly appreciated and the text about these topics is pertinent and can elucidate some deep and insightful understanding in this field.

Some suggestions could be considered by the authors. First, the authors have already mentioned that apart from China and the United States, cooperation among other countries is limited. We think that authors could call for increased inter-country cooperation to break down academic barriers to promoting the development of this research field. Then, the span of journal impact factor levels is very large, from *Frontiers in Oncology* and *Cancers* to *Nature* and *Cell*, which indicates that the depth of relevant research spans a wide range, and the articles published in *Nature* or *Cell* could indicated that autophagy is critically important in HCC with great fundamental and translational value, and can promote the disciplinary integration, involving scientists from a wider range of fields than just those related to hepatology or autophagy.

In addition, we noted an interesting phenomenon that there was only one review article included in the top 10 cited manuscripts^4^, which is inconsistent with the common understanding of citation in scientific research^5^. Here, we speculated that since autophagy in HCC is an emerging topic born around 2013, scientists are now focusing more on the signaling pathways and targets involved based on original studies instead of review articles. However, we would advise authors to add some comments about this slightly anomalous phenomenon in the discussion part, for example, elaborating on the contribution of the top 10 cited original articles to this research topic.

Moreover, the total citations (TCs) about manuscripts were calculated, and we suggest that the average annual citations (AACs) per year is also needed because the TCs are determined by AAC and the time from first publication to this analysis. The AAC can better dynamically reflect the influence of a manuscript, which could be presented in Table 5, or authors can add another table to show the top 10 manuscripts ranked by AACs. Additionally, the ACCs were estimated in journals and institutions; however, the arithmetic mean of citation is strongly influenced by a minority of highly cited papers and does not adequately reflect the ‘average’ citation rate of a particular journal or institution. Currently, the median value of citations (MVCs) was suggested to be more appropriate for expressing citedness^6^. Authors could try to analyze the MVC and combine it with AAC and TC to more precisely and rigorously reflect the ‘average’ citation rate of journals and institutions.

In conclusion, we want to express much appreciation to He and colleagues for writing this creative, well-designed, and inspiring paper. However, we simply make some comments from a methodological and results perspective that might help further improve the work.

## Ethical approval

Not applicable.

## Consent

Not applicable.

## Sources of funding

Not applicable.

## Author contribution

S.H.: conceptualization and writing of the original draft; H.Z.: review, editing, and supervision.

## Conflicts of interest disclosure

The authors declare that they have no conflicts of interest.

## Research registration unique identifying number (UIN)

Not applicable.

## Guarantor

Both authors.

## Data availability statement

Not applicable.

## Provenance and peer review

Not applicable.
